# An empirical study of mHealth adoption in a developing country: the moderating effect of gender concern

**DOI:** 10.1186/s12911-016-0289-0

**Published:** 2016-05-03

**Authors:** Md Rakibul Hoque

**Affiliations:** Department of Management Information Systems, University of Dhaka, Dhaka, 1000 Bangladesh; School of Management, Huazhong University of Science and Technology, Wuhan, 430074 China

**Keywords:** mHealth, Developing country, Adoption

## Abstract

**Background:**

mHealth has become a valuable tool for providing health care services in developing countries. Despite the potential benefits of mHealth, its adoption remains a very challenge in developing countries like Bangladesh. The aim of this study is to investigate the factors that affect the adoption of mHealth services in Bangladesh using Extended Technology Acceptance Model (TAM).

**Methods:**

Data were collected from over 250 respondents in Dhaka, Bangladesh. The data were analyzed using the Partial Least Squares (PLS) method, a statistical analysis technique based on the Structural Equation Modeling (SEM).

**Results:**

The study found that perceived ease of use, perceived usefulness and subjective norm (*p* < 0.05) had significant positive impact on the intention to adopt mHealth services. Surprisingly, the effects of personal innovativeness in IT (*p* > 0.05) on mHealth adoption were insignificant. This study also revealed that gender was strongly associated with the adoption and use of mHealth in developing countries.

**Conclusions:**

The findings of this study can be used by government, policy makers, and mobile phone Company to maximize the acceptance of mHealth services in Bangladesh. The paper concludes with a discussion of research results and draws several implications for future research.

## Background

Over the past decade, the rapid advances in mobile and wireless technologies have paved the way to transform health service delivery across the world. The unprecedented spread of mobile technologies and their innovative application to address health priorities has developed a new field of e-Health, known as mHealth [1]. mHealth is defined as “the application of wireless technologies to transmit different data contents and services which are accessible by health workers through mobile devices such as mobile phones, PDAs, smart phones and Tablet PCs” [2]. It provides medical and health care services to both health professionals and users. The work of health professional (i.e doctors, nurses) may be better supported and user may obtain useful information and guidance to manage their health better by mHealth [3]. Moreover, mHealth has transformed the healthcare in developing countries by serving the unserved [4].

Wireless technologies and mobile phones have reached far more people than any other new advanced information and communication technologies, especially in developing countries. Although the use of internet has also increased extensively in recent years, about 31 % of the people in developing countries had internet access in 2013, whereas nearly 80 % had mobile phone subscriptions [5]. A World Health Organization (WHO) report in 2011 suggested that mobile phone coverage had reached 89 % of all Bangladeshi adults [6]. At the end of the December 2014, the total number of Mobile Phone subscribers has reached 120.350 million in Bangladesh [7]. Bangladesh Demographic and Health (DHS) surveys of 2007 and 2011 showed that household mobile phones ownership has increased from 32 to 78 %, varying from 75 % in rural areas to 89 % in urban [8, 9]. In addition, Bangladesh claimed to have the cheapest mobile phone call rates in the world [10]. Therefore, it is the golden opportunity for Bangladesh to develop mobile health services for their citizen so that they could better manage their health. Through mobile health services, user can seek medical advice; make appointment with doctors; access medical test result; and access to personal health information [11].

In recent years, the number of chronic disease among younger citizen is gradually increasing due to different factors such as smoking, intensive academic and social pressure [12]. More than 70 % world’s smokers, most of them are young, are living in just ten countries and Bangladesh is one of them. In Bangladesh, more than 50 % of younger males and 30 % of younger females consume tobacco in any form, smoking or smokeless [13]. In developing countries like Bangladesh, smoking is the main causes of different diseases which lead to premature death for young citizen. Young citizen in Bangladesh also suffer from hypertension and Type 2 diabetic which are considered to be two of the leading causes of death worldwide and risk factors for strokes, heart attacks, heart failures, cardiovascular diseases and coronary artery diseases [14].

Mobile phones based health services, commonly known as mHealth, provides personalized and tailored health care services to those who need it, especially young citizen [11]. It has been regarded as best tools for curing diseases and improving health condition [15, 16]. mHealth services leads people to manage chronic disease more effectively and improve their quality of life. It can prevent and control hypertension and Type 2 diabetic. It has been also gained popularity for smoking cessation for younger citizen in developing countries [17, 18]. mHealth intervention can reduce cost, better reach, increased interaction between patients and doctors and easier as well as faster to send messages regarding the diseases and health. Adoption of mHealth can improve health, food intake, exercise, sleep, blood sugar and other physiological states and behaviors.

Although there have been considerable benefits of mHealth services, they have encountered numerous challenges and difficulties as a newly emerging phenomenon [19]. In Bangladesh, this service is still in the infancy stage and requires extensive research on user adoption process, especially considering users who have vast knowledge (i. e young citizen) about using mobile or smart phone. However, very few to no studies consider the adoption of mHealth services with regard to young citizen in developing countries. Therefore, factors that influences the adoption of mHealth services by younger citizen must be investigated - a population that is most favorably placed in the adoption of innovations and technology. It is also important to consider the gender issues in mHealth adoption because the gender gap still exists in developing countries. Research found that female is less interested than male to use new technology, a ‘gender gap’ almost twice that for all low- and middle-income countries in South East Asia [20]. So, this study also includes gender as a moderating variable. The objective of this study is to identify the factors that influence the adoption of mHealth services among younger people in Bangladesh.

## Theoretical framework

**Fig. 1:**
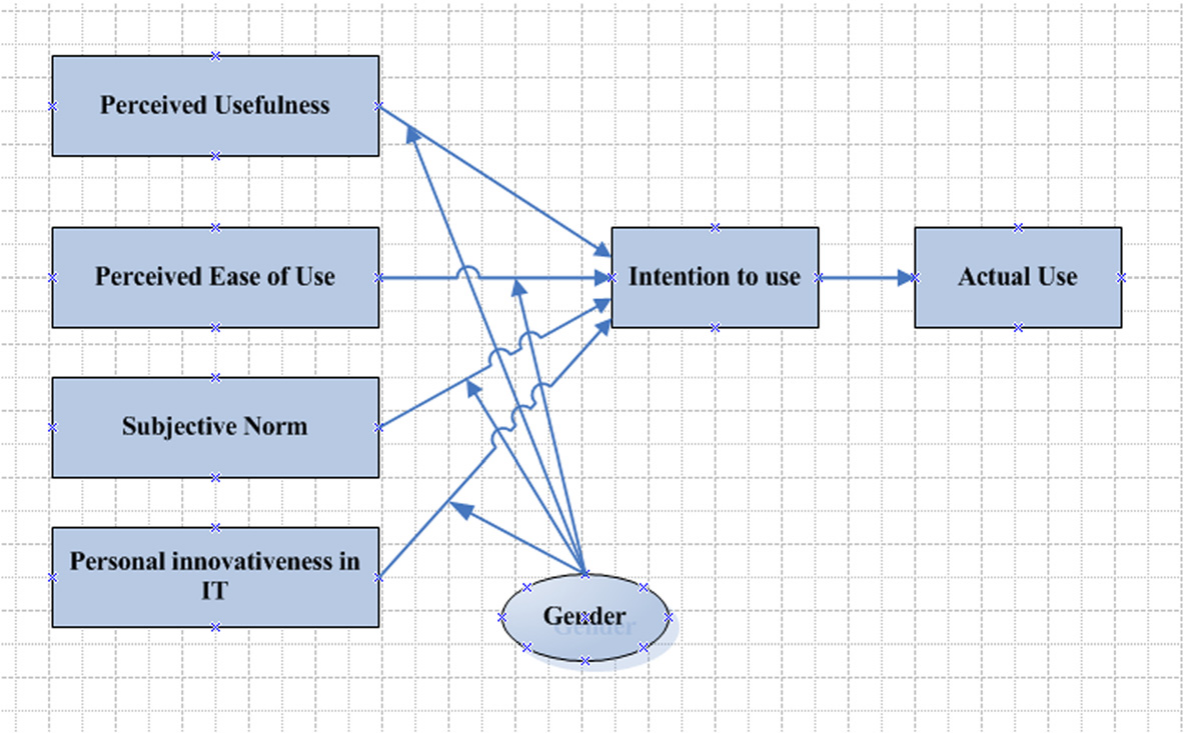
Research model

There are different theories of technology acceptance namely the theory of planned behavior (TPB), innovation diffusion theory (IDT), theory of reasoned action (TRA), technology acceptance model (TAM) and theory of innovation Adoption (TIA). Among them, the TAM is the most influential model for technology adoption. Though the basic TAM presents a rigorous explanation in predicting the user’s acceptance of technology, some studies suggest that additional explanatory variables may be needed depending on the specific technology context [21]. Therefore, this study extends the TAM (shown in Fig. 1) with two additional salient variables, subjective norm and personal innovativeness in IT, to enhance prediction of intention to use mHealth in Bangladesh context. Gender was included in the proposed model as factors to moderate the effect of dependent and independent variable.

The TAM hypothesizes that the most important determinant of actual technology use is the intention to use the technology, whereas intention in turn, is predicted by perceived usefulness (PU) and perceived ease of use (PEU) [22]. TAM has received much attention from researchers and considered as a prevailing model for predicting usage intention and acceptance behavior [23]. Since the introduction of TAM, a number of studies have applied it to predict user acceptance of variety IT applications such as wireless internet [24], online shopping [25], email and voice mail [26], e-Health [27], clinical information systems [28] and mHealth [29, 30].

## Perceived Usefulness (PU)

Perceived usefulness is defined as “the degree to which a person believes that use of the system will enhance his or her performance” [31]. Many existing studies have demonstrated that perceived usefulness positively influences behavioral intention to use a new technology such as online banking [32], e-ticketing [33], PDA [34], e-learning [35], mobile learning [36], online system [37] and mobile services [38]. In the context of mHealth, researchers found that PU is a vital factor which determines the adoption of mHealth since users consider its benefits [39, 40]. Based on the above literature, we proposed the following hypotheses:

1. : *Perceived Usefulness has a positive influence on the intention to use mHealth services among younger in Bangladesh*.

## Perceived Ease of Use (PEU)

Perceived ease of use is defined as “the degree to which a person believes that using the system will be free of effort” [31]. The researchers have suggested that ease of use is a major attribute of mHealth applications [41], electronic health record [42], mobile banking [43], e-government [44], and online banking [45]. Consequently, the following hypothesis is suggested:

H2 : *Perceived ease of use has a positive influence on the intention to use mHealth services among younger in Bangladesh.*

## Subjective norm

Subjective norm is defined as “person’s perception that most people who are important to him think he should or should not perform the behavior question” [46]. Existing research on information systems found a significant relationship between subjective norm and adoption of IS [47]. The researcher has suggested that subjective norm positively influence the intention to use electronic brokerages service [48], online services [49], e-payment [50], mobile banking [51], internet banking [52], instant messaging [53] and mHealth [54]. Consequently, the following hypothesis is suggested:

H3 : *Subjective norm has a positive influence on the intention to use mHealth services among younger in Bangladesh*.

## Personal innovativeness in IT

Personal innovativeness is defined as “the degree to which an individual is relatively earlier in adopting new ideas” [55]. Agarwal and Prasad [56] empirically tested the influence of personal innovativeness in IT on information technology use. Many existing studies have demonstrated that personal innovativeness in IT significantly influences the intention to use a new technology such as internet [57], public grid computing [58], PDA [59], information systems [60] and mHealth [54]. Consequently, the following hypothesis is suggested:

H4 : *Personal innovativeness in IT has a positive influence on the intention to use mHealth services among younger in Bangladesh.*

The relationship between intention to use and actual use of technology in TAM model are also empirically examined in different studiest. The variable of “Actual Use” (AU) has been measured, by intention to use [61]. Turner et al. [62] conducted a systematic literature review on TAM based on 6 digital libraries. They found that BI is highly correlated with actual usage of 79 relevant empirical studies. Legris [63] confirmed that intention to use influence the actual use of technology. Horton et al. [64] found that higher the intention to use, the higher the actual use of intranet. BI–AU relationships are also tested by Lim et al. [30] in their study of mHealth adoption. Therefore, we propose the following hypothesis:

H5 : *Intention to use has a positive influence on actual use of mHealth services among younger in Bangladesh*.

## The moderating role of gender concern

Gender, as a moderating factor, received less consideration in past technology adoption research compared with other factors such as age, culture and experience. Although gender are considered as an important factor in technology adoption, original TAM makes no references of gender differences and genders are not examined in IT acceptance model [65]. Generally, male has less anxious and more positive attitudes about technology innovations [66]. Wang et al. [67] explored that gender differences moderate the effects of learning on m-learning use intention. Gefen and Straub [25] found that male and female differ in their perception of e-mail use. Van et al. [68] suggest that man and woman would differ in their acceptance of web site and web-based shopping. Ong and Lai [69] have suggested that male’s score on the PU and PEU of e-learning is higher than scores of female. Therefore, we proposed the following hypotheses:

H6 : *Gender has a significant moderating role in the relationship between PU and intention to use mHealth services among younger in Bangladesh.*
H7 : *Gender has a significant moderating role in the relationship between PEU and intention to use mHealth services among younger in Bangladesh.*
H8 : *Gender has a significant moderating role in the relationship between subjective norm and intention to use mHealth services among younger in Bangladesh.*
H9 : *Gender has a significant moderating role in the relationship between personal innovativeness in IT and intention to use mHealth services among younger in Bangladesh.*

## Methods

### Research setting

The target population for this study was the younger citizen in Bangladesh. This study used convenience sampling method as survey instrument. Convenience sampling is “a type of nonprobability sampling which involves the sample being drawn from that part of the population which is close to hand” [70]. Moreover, it is cost effective and has been widely used in (Information Systems (IS) research [71]. The sample was taken from younger citizens in Dhaka City of Bangladesh, who were students at the University of Dhaka. They were considered to be suitable because of their comfortability with the use of technology for activities such as searching information, gaming in addition to simple communication. Prior to commencing the research, ethical approval was sought and obtained from the Center for Modern Information Management, School of Management, Huazhong University of Science and Technology, Wuhan, China. The author also contacted with the chairman of MIS department at University of Dhaka to seek permission to collect data from students. All participants in the research were given consent forms and information sheets which clearly explained the purpose of the study. Respondents were also made aware of their rights to withdraw participation at any time during the study. Respondents were also made aware of the fact that they may request the findings of the research once it is completed. The survey was conducted in November 2014.

### Measurement

All items in this study were adopted from previous studies. The items were modified to match the context of this study related to mHealth in the context of Bangladesh. PU and PEU were measured with four items adapted from Davis [22], Chau & Hu [72]. Subjective norm with three items was adapted from Wu and Chen [73]. Personal innovativeness in IT with three items was adapted from Agarwal and Prasad, [56], Yi et al. [74]. Intention to use with three items was adapted from Venkatesh et al. [46], Davis [22]. Finally, actual use with three items was adapted from Taylor and Todd [75], Davis and Venkatesh [76]. The details of the measurement items and the source of literature for each construct are presented in Table 1.

**Table 1:** Measurement items

Items	References
Perceived Usefulness	
PU1: Using the mHealth services will improve my life quality	Davis (1989) [22, 31], Chau & Hu (2002) [72]
PU2: Using the mHealth services will make my life more convenient	
PU3: Using the mHealth services will make me more effective in my life	
PU4: Overall, I find the mHealth services to be useful in my life	
Perceived Ease of Use	
PEU1: Learning to operate the mHealth services will be easy for me	Davis (1989) [22, 31], Chau & Hu (2002) [72]
PEU2: I can easily become skillful at using the mHealth services	
PEU3: I can get the mHealth services to do what I want it to do	
PEU4: Overall, the mHealth services are easy to use	
Subjective Norm	
SN1. My close friends think that I can use mHealth	Wu and Chen (2005) [73]
SN2. My close friends think that I should use mHealth	
SN3. My close friends think that I must use mHealth	
Personal innovativeness in IT	
PIIT1: If I heard about a new information technology, I would look for ways to experiment with it.	Agarwal and Prasad, (1998) [56], Yi et al. (2006) [74]
PIIT2: Among my peers, I am usually the first to try out new information technologies.	
PIIT3: In general, I am not hesitant to try out new information technologies.	
Intention to Use	
INT1: I have high intention to use the mHealth service	Venkatesh et al. (2003) [46], Davis (1989) [22, 31].
INT2: I intend to learn about using mHealth services	
INT3: I plan to use mHealth services to manage my health	
Actual Use	
ACT1: mHealth service is a pleasant experience	Taylor and Todd (1995) [75], Davis and Venkatesh (2004) [76].
ACT2: I use mHealth service currently	
ACT3: I spend a lot of time on mHealth service	

### Questionnaire design and data collection

The data for this study was collected through a structured questionnaire consisting of two parts. Part A contains the demographic information, while Part B includes previously validated questionnaires for the different constructs. The items of the construct were measured using a 5-point Likert, with answer choices ranging from (1) “strongly disagree” to (5) “strongly agree”. The questionnaires were distributed among 250 respondents in Dhaka. Out of 250 questionnaires, 234 were returned to the researcher. However, 7 incomplete questionnaires were excluded from the study.

### Data analysis

Data from questionnaires were inserted into Microsoft excel and imported into SmartPLS software, a technique of Structural Equation Modeling (SEM), for statistical analysis. Structural Equation Modeling is widely accepted paradigm to gauge the validity of meaty theories with empirical data. It is an extensive statistical representation of general linear modeling. One of the notable applications of SEM is that it can be applied to explore out the relationships among latent constructs and which are indicated by multiple measures. SEM is composed of two the evaluation of twin models: measurement model and a path model. Path models is an extensive form of multiple regression model in which various multiple regression are estimated simultaneously [77]. In other words, path analysis can be regarded as a special case of SEM in which the structural relations among latent variables are molded.

## Result

### Demographic information

Table 2 shows the demographic characteristics of the respondents. There is no large gap between male and female respondents (59 and 41 %). About 57 % of the respondents’ ages are between the 20 and 30, half of them are less than 20 (27 %). Majority of the respondents (86 %) had less than 6 years of IT experiences, while only 3 % had more than 10 years of IT experiences. Around 42 % had Master’s degree and 26 % had Bachelor degree.

**Table 2:** Demographics of respondents

Descriptions		Frequency	Percentage
Gender	Male	133	59 %
	Female	94	41 %
Educational Qualification	Bachelor	59	26 %
	Masters	96	42 %
	Others	72	32 %
Age	Less than 20	61	27 %
	20–30	129	57 %
	More than 30	37	16 %
IT experience	Less than 1 years	22	10 %
	1–3 years	68	30 %
	4–6 years	105	46 %
	7–9 years	26	11 %
	More than 10 years	6	3 %

### Measurement model

The validity and reliability of the measures should be examined before testing the hypothesis [78]. The reliability was evaluated by considering Cronbach’s alpha and composite reliability. The reliability is considered to be satisfactory when composite reliability and Cronbach’s alpha have value greater than 0.70. Convergent validity is considered to be satisfactory when measurement constructs have an average variance extracted (AVE) of at least 0.50 and items loading are well above 0.50 [79]. Table 3 presents the composite reliability, Cronbach’s alpha and average variance extracted (AVE), while Table 4 shows the item loading.

**Table 3:** The measurement model

Constructs	CR	Cronbach’s Alpha	AVE
Actual Use	0.9200	0.8693	0.7935
Intention to Use	0.8932	0.8198	0.7364
Perceived Ease of Use	0.9176	0.8803	0.7361
Perceived Usefulness	0.8911	0.8380	0.6719
Personal innovativeness in IT	0.9449	0.9335	0.8511
Subjective Norm	0.9031	0.9355	0.7582

*AVE* average variance extracted, *CR* composite reliability

**Table 4:** Cross-loading matrix

	ACT	INT	PEU	PI	PU	SN
ACT1	**0.8289**	0.6314	0.6236	0.2337	0.4696	−0.1106
ACT2	**0.9119**	0.7525	0.7417	0.1027	0.5053	−0.1182
ACT3	**0.9283**	0.7993	0.795	0.1321	0.5379	−0.1969
INT1	0.6533	**0.8102**	0.6511	0.058	0.4363	−0.0398
INT2	0.6735	**0.8519**	0.6671	0.0824	0.4044	−0.0084
INT3	0.7822	**0.9095**	0.7347	0.1032	0.487	−0.148
PEU1	0.8053	0.7592	**0.8788**	0.0945	0.5026	−0.1314
PEU2	0.6453	0.6346	**0.8516**	0.1239	0.4231	−0.1523
PEU3	0.5912	0.599	**0.8033**	−0.0268	0.3111	−0.0417
PEU4	0.7262	0.7299	**0.8954**	−0.0484	0.4602	−0.0478
PI1	0.0896	−0.0216	−0.0424	**0.8940**	0.4666	−0.8265
PI2	0.1321	0.0492	−0.0118	**0.9133**	0.4903	−0.7864
PI3	0.1598	0.0868	0.0492	**0.9592**	0.4969	−0.8178
PU1	0.4876	0.4607	0.4625	0.3904	**0.8194**	−0.3841
PU2	0.3983	0.3387	0.3405	0.3927	**0.7878**	−0.4056
PU3	0.4658	0.4253	0.4378	0.5039	**0.8303**	−0.5416
PU4	0.4942	0.4512	0.3865	0.4335	**0.8402**	−0.455
SN1	−0.1425	−0.037	−0.0824	−0.8696	−0.488	**0.8423**
SN2	−0.1018	−0.0485	−0.0644	−0.7905	−0.5011	**0.9776**
SN3	−0.0369	0.0282	0.0007	−0.8093	−0.4093	**0.7806**

It is apparent from the Table 3 that the Cronbach’s alpha values ranged from 0.81 to 0.93, and composite reliability ranged from 0.89 to 0.94 which indicates adequate internal reliability. Item loading, ranged from 0.78 to 0.97 and AVE, ranged from 0.67 to 0.85, are greater than the recommended level. Therefore, conditions for convergent validity were met.

On the other hand, the discriminant validity was examined by the square root of the AVE and cross loading matrix. The square root of the AVE of a construct must be larger than its correlation with other construct for satisfactory discriminant validity [80]. What is interesting in this table is that the square roots of AVE, shown in Table 5, were greater than their corresponding correlation, representing that our data had good discriminant validity.

**Table 5:** Correlation matrix and square root of the AVE

	ACT	INT	PEU	PI	PU	SN
ACT	**0.8907**					
INT	0.8218	**0.8581**				
PEU	0.8134	0.7986	**0.8579**			
PI	0.1693	0.0956	0.0423	**0.9225**		
PU	0.5671	0.5168	0.5005	0.5251	**0.8197**	
SN	−0.1620	−0.0798	−0.1094	−0.8433	−0.5448	**0.87075**

### The structural model

The structural model was constructed to identify the path direction and strength of relationships among the latent variable in the research model. Bootstrapping method was used to test the hypothesis. First, we tested the relationship between endogenous and exogenous variable. Then, we tested the moderating effect of gender. Table 6 showed the path coefficient (*β*) and *t*-statistics. It was found that PU (*t* = 4.0398, *β* = 0.2095), PEU (*t* = 15.5538, *β* = 0.7005) and subjective norm (*t* = 2.2041, *β* = 0.2207) had significant effect on intention to use mHealth adoption, while personal innovativeness in IT (*t* = 1.2845, *β* = 0.1216) had no significant effect on intention to use mHealth. The study also found that intention to use mHealth (*t* = 0.8228, *β* = 51.2824) had significant effect on actual use of mHealth. Therefore, among the primary hypothesis, H1, H2, H3, and H5 were supported, whereas H4 was not supported. From the table, it can be stated that the model explains 67.5 % of the variance in intention to use mHealth (R^2^ = .675) and 65.5 % of variance in actual use of mHealth (R^2^ = .655).

**Table 6:** Structural model

Path	*β*	*t* Statistics	Comments
INT - > ACT	0.8228	51.2824	Supported
PEU - > INT	0.7005	15.5538	Supported
PI - > INT	0.1216	1.2845	Not Supported
PU - > INT	0.2095	4.0398	Supported
SN - > INT	0.2207	2.2041	Supported

### The moderating effect of gender

This study found a significant moderating effect of gender on mHealth adoption. Data from the table illustrates the information that male have a higher level of mHealth adoption intention than females in terms of PEU (0.6556 versus 0.1445, *t* = 3.784), PI (0.6058 versus 0.0749, *t* = 3.334), and SN (0.8584 versus -0.3763, *t* = 9.512). Thus, our Hypotheses 7–9 were all supported, and we can conclude that male find it easy to adopt mHealth. But, the aspect of PU (0.3244 versus 0.0140, *t* = 2.104), female have a higher level of mHealth adoption intention than males (Table 7).

**Table 7:** Moderating effect of gender

	Male		Female		Comparison
Path	*β*	*t*-statistics	*β*	*t*-statistics	*t*-statistics
PEU - > INT	0.6556	5.8639	0.1445	1.6618	3.784
PI- > INT	0.6058	5.4781	0.0749	0.7133	3.334
PU - > INT	0.0140	0.5374	0.3244	2.6782	2.104
SN - > INT	0.8584	9.8919	−0.3763	4.2134	9.512

## Discussion

This study extended TAM model and supported it using empirical data in the context of mHealth adoption in Bangladesh. Regarding TAM related variables, the result shows that both PEU and PU had significant influence on intention to use mHealth. This finding is consistent with the existing literature on the topic that adoption of a system such as mHealth is dependent on usefulness of the systems and how easy it is to use it [81]. Teo et al. [82] also demonstrated that PU and PEU to be significant determinant of behavioural intention to use technology. This study also infers that intention to use found to be a stronger predictor of actual use of mHealth.

In addition to the TAM variables, our results show the strong positive relationship between subjective norm and intention to use mHealth in Bangladesh. Many previous researchers theorize that subjective norm has a significant effect on intention to use a system [83]. We can conclude that opinions from family members and friends affect the decision to adopt mHealth because it is relatively new applications in Bangladesh. We suggest that Telecom Company and healthcare provider can invest money and effort for promotional activities on virtual communities.

However, the results of this current study indicate that Personal innovativeness in IT has less significant effect on mHealth adoption. This finding is surprising because many previous studies confirm the relationship between Personal innovativeness in IT and technology adoption [84]. Our finding could be reflective on the fact that more innovative people do not necessarily have more intention to use technology. Another possible explanation is that that younger people in Bangladesh are not concerned about mHealth apps, although they are the user of different mobile apps. Therefore the Personal innovativeness in IT does not play a significant role in the mHealth adoption in the context of Bangladesh. Further study is needed to advance our understanding of Personal innovativeness in IT.

This study found the moderating effect of gender differences. In our study men and women were responded differently in their concern about PU, PEU, subjective norm and Personal innovativeness in IT of mHealth adoption. Venkatesh and Morris [85] indicated that compared to female, male's technology adoption decisions were more strongly influenced by their perceptions of usefulness. In contrast, female were more strongly influenced by perceptions of subjective norm and ease of use. According to the technology adoption research, male and female react differently due to their differences toward technology adoption [69].

## Study limitations, future directions and conclusion

There are some limitations in this study. First, we surveyed only Dhaka City, urban areas in Bangladesh which may raise concern about the generalizability of the findings. Future research should give more attention to rural areas in Bangladesh. Future research should give more attention to rural areas in Bangladesh. Second, we surveyed only younger citizens in Bangladesh. The paper does not engage with the factors that influence the adoption of mHealth. Future research could investigate the mHealth adoption among other age group such as elderly. Finally, this study used convenience sampling method as survey instrument which may not be the representative of the entire population and results may be biased.

Although, there are some limitations, this study is the milestones and future scope for researchers in developing countries to understand the factors that influence the adoption of mHealth. This study extended TAM model to understand the factors affecting mHealth adoption in developing countries. Our findings showed that extended TAM is a valid model to understand the adoption of mHealth in developing countries. Identified factors in this study can provide necessary assistants to the development and adoption of mHealth. Successful adoption of mHealth depends on the engagement of patient, telecom industry, healthcare organization, app developer, researcher and other stakeholders. App developer and designers should design mHealth that are easy to use. Healthcare organization and telecom industry can provide adequate training and support during implementation and use of mHealth. Government should provide a facilitating environment where citizens are encouraged to use new technologies such as mHealth.

## Ethics approval and consent to participate

Prior to commencing the research, ethical approval was sought and obtained from the Center for Modern Information Management, School of Management, Huazhong University of Science and Technology, Wuhan, China. The author also contacted with the chairman of MIS department at University of Dhaka to seek permission to collect data from students. All participants in the research were given consent forms and information sheets which clearly explained the purpose of the study. Respondents were also made aware of their rights to withdraw participation at any time during the study. Moreover, this study did not include any minors or vulnerable adults and therefore, no major or special ethical issues were involved in this study. The questionnaire also provided respondents with information on how to lodge a complaint with Ethics committee should they feel a need to do so. Respondents were also made aware of the fact that they may request the findings of the research once it is completed.

## Consent for publication

Not applicable.

## Availability of data and material

The specific data used in this study is available upon request from the authors.

## Abbreviations

ACT: Actual Use
AVE: Average Variance Extracted
CR: Composite Reliability
IDT: Innovation Diffusion Theory
INT: Intention to Use
PEU: Perceived Ease of Use
PIIT: Personal Innovativeness in IT
PLS: Partial Least Squares
PU: Perceived Usefulness
SEM: Structural Equation Model
SN: Subjective Norm
TAM: Technology Acceptance Model
TIA: Theory of Innovation Adoption
TPB: Theory of Planned Behavior
TRA: Theory of Reasoned Action
WHO: World Health Organization
